# Coexistence of atypical hemolytic uremic syndrome and crescentic IgA nephropathy treated with eculizumab: a case report 

**DOI:** 10.5414/CNCS108889

**Published:** 2016-12-12

**Authors:** Daisuke Matsumura, Atsushi Tanaka, Tsukasa Nakamura, Eiichi Sato, Koichi Node

**Affiliations:** 1 Division of Nephrology, Department of Internal Medicine, Shinmatsudo Central General Hospital, Matsudo, and; 2 Deaprtment of Cardiovascular Medicine, Saga University, Saga, Japan

**Keywords:** acute kidney injury, atypical hemolytic uremic syndrome, crescentic IgA nephropathy, eculizumab, end-stage renal disease, plasma exchange

## Abstract

Rapid progression to end-stage renal disease has been reported in a minority of patients with immunoglobulin A (IgA) nephropathy. In particular, crescentic IgA nephropathy has a poor prognosis in patients with a higher initial serum creatinine level. The complement system plays an important role in the pathogenesis of crescentic IgA nephropathy. Atypical hemolytic uremic syndrome (aHUS), which is characterized by thrombotic microangiopathy, is distinct from Shigatoxin-induced HUS and thrombotic thrombocytopenic purpura. aHUS is associated with dysregulation of the alternative complement system. Eculizumab, an anti-C5 antibody, is effective in limiting complement activation in patients with paroxysmal nocturnal hemoglobinuria, aHUS, or refractory IgA nephropathy in some case reports. We herein report the case of a 42-year-old man with acute kidney injury (AKI) clinically and histologically diagnosed with the coexistence of aHUS and crescentic IgA nephropathy. The patient was treated with steroids, plasmapheresis, and hemodialysis; however, eculizumab treatment was initiated on hospital day 21 due to resistance to and dependence on the conventional aggressive therapy. Clinical remission of aHUS was achieved on day 70, but the renal function failed to recover from dialysis dependence. To the best of our knowledge, this is the first report showing the clinical course of a refractory patient with the coexistence of aHUS and crescentic IgA nephropathy treated with eculizumab. This case highlights the clinical importance of early diagnosis and appropriate initiation of eculizumab for the treatment of this type of AKI.

## Introduction 

Hemolytic uremic syndrome (HUS) is defined by the triad of mechanical hemolytic anemia, thrombocytopenia, and renal dysfunction [1]. Atypical HUS (aHUS) does not include diarrhea and is associated with defective complement regulation, resistance to treatment, and poor prognosis [2]. Eculizumab, an anti-C5 antibody, is effective in limiting complement activation in patients with aHUS and has recently become a therapeutic option for aHUS [3]. Investigators have published case reports describing the effectiveness of eculizumab and plasma exchange in the treatment of patients with aHUS [3, 4]. Crescentic IgA nephropathy has a poor prognosis, and the initial serum creatinine level may predict renal failure [5]. Early initiation of eculizumab in patients with progressive IgA nephropathy may have a beneficial effect by blocking complement-mediated renal inflammation [6]. Crescentic IgA nephropathy causing end-stage renal disease (ESRD) coexisting with aHUS has rarely been reported, and the effectiveness of eculizumab in such patents is still unknown. Here, we are the first to present a case with clinical and pathological features of aHUS and crescentic IgA nephropathy with ESRD treated with steroids, plasma exchange, and eculizumab. 

## Case history 

**Figure 1.**
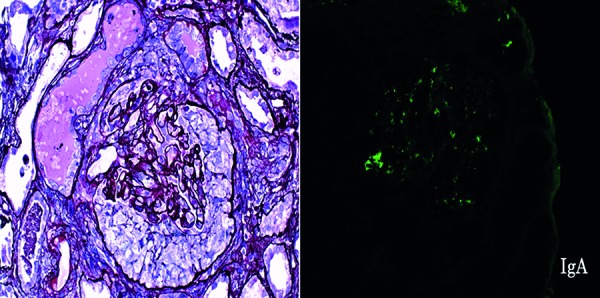
Left panel: PAM staining showing crescent formation (×400). Right panel: Immunofluorescence demonstrating immunoglobulin A (IgA) deposition (×400).

**Figure 2.**
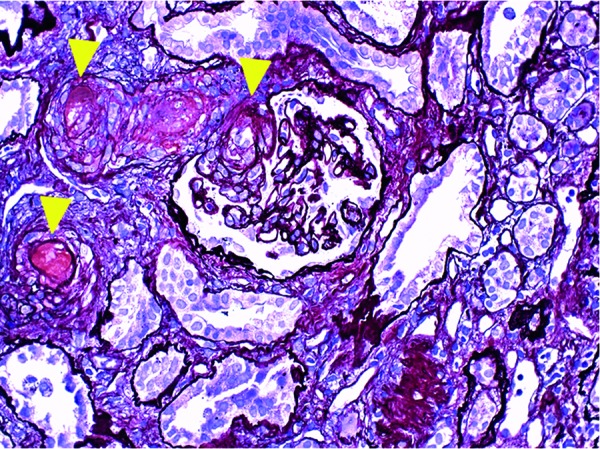
PAM staining showing thrombi (arrowheads) in the periglomerular capillary tuft and vascular pole (×400).

A 43-year-old man was admitted to our hospital after suffering from 10 days of nausea, diarrhea, pretibial edema, and low-grade fever. Since the patient did not undergo annual routine medical examinations, medical data from the past 20 years were unavailable. There was no family history of kidney disease, cardiovascular disease, stroke, or clotting disorders. A physical examination revealed the following: body height 173 cm, body weight 66 kg, body temperature 37.3 °C, blood pressure 160/90 mmHg, and heart rate 110 beats/min. His skin looked pale and slightly icteric. Laboratory data revealed severe anemia (hemoglobin 7.7 g/dL), platelet count 5.4 × 10^4^/µL, lactate dehydrogenase (LDH) 1,342 IU/L, total bilirubin 4.5 mg/dL, elevated D-dimer 4.7 µg/mL, urinary protein excretion 570 mg/dL, urinary blood cells over 100 cells/high power field, many urinary blood cell casts and granular casts/high power field, urinary N-acetyl-β-D-glucosaminidase (NAG) enzyme 151 IU/L (normal range: 0 – 11.4 IU/L), β2-microglobulin (MG) 5,008 µg/L (normal range: 0 – 11.4 µg/L), urinary liver type fatty acid binding protein (L-FABP) 273.2 µg/g creatinine (Cr) (normal range: 0 – 8.4 µg/g×Cr), serum Cr 18.78 mg/dL, blood urea nitrogen (BUN) 121 mg/dL, CH50 22.2 /mL (normal range: 25 – 48 /mL), C3 49.6 mg/dL (normal range: 65.0 – 135.0 mg/dL), C4 9.6 mg/dL (normal range: 13.0 – 35.0 mg/dL), IgG 610 mg/dL (normal range: 870 – 1,700 mg/dL), IgA 290 mg/dL (normal range: 110 – 410 mg/dL), and IgM 49 mg/dL (normal range: 46 – 260 mg/dL). Autoantibody to complement factor H, antinuclear antibodies, anti-DNA antibody, myeloperoxidase anti-neutrophil cytoplasmic antibody (MPO-ANCA), proteinase 3 anti-neutrophil cytoplasmic antibody (PR3-ANCA), and anti-glomerular basement membrane (GBM) antibody were negative. Serologic markers for human immunodeficiency virus, hepatitis B virus, and hepatitis C virus were negative. Coombs test and stool culture for Shiga toxin-producing *Escherichia coli* were negative. ADAMTS13 (a disintegrin-like and metalloprotease with thrombospondin type 1 motif 13) activity was 60%. There were increased schistocytes (10%) on the peripheral smear. The ultrasound showed kidney atrophy (left: 92 mm × 36 mm × 44 mm, right: 94 mm × 34 mm × 42 mm) without abnormal echogenicity, presumably indicating prolonged existence of renal dysfunction. On the day of admission, a femoral catheter was placed and he underwent a session of hemodialysis. On day 5, a percutaneous renal biopsy was performed. The biopsy section contained 20 glomeruli: 5 globally-sclerosed glomeruli, 3 with segmental sclerosis, 7 with cellular crescent formation, 2 with fibrocellular crescent formation, and 3 with mesangial hypercellularity with IgA and C3 deposition (Figure 1). Some glomeruli revealed evidence of progressive thrombotic microangiopathy (TMA) with excessive neutrophil infiltrates, capillary loop occlusions, abnormal capillary loop thickening, and thrombi filling portions of the peri-glomerular capillary tuft and vascular pole (Figure 2). Thus, these clinical and histological features characterized both aHUS and crescentic IgA nephropathy. The patient was treated with intravenous methylprednisolone 1 g daily for 3 days, followed by oral prednisolone 40 mg daily. Plasma exchange with fresh plasma was initiated on day 10 (60 mL/kg of fresh frozen plasma for replacement) and performed twice a week, in addition to hemodialysis 3 times a week. However, because the renal failure persisted, the patient agreed to receive eculizumab treatment to rescue renal function and was given meningococcal vaccination for initiation of eculizumab on day 21 [7, 8]. Eculizumab was continued at 900 mg every week for a total of 4 weeks and then transitioned to 1,200 mg every 2 weeks. The serial changes in the laboratory values are shown in Table 1. On day 70, the hemoglobin level, platelet count, CH50, LDH, total bilirubin, and D-dimer had almost returned to the normal range. In addition, the relevant biomarkers such as urinary protein excretion, L-FABP, NAG, and β2-MG were also reduced, but remained higher than the normal range. Unfortunately, the serum creatinine level remained elevated (7.22 mg/dL) at day 70, and hemodialysis was continued. 

## Discussion 

We herein reported a patient with crescentic IgA nephropathy and aHUS treated with steroids, plasma exchange, and eculizumab. The hemolysis resolved, but the renal dysfunction persisted. The initial diagnosis of his renal dysfunction was thought to be acute kidney injury (AKI) caused by aHUS. In general, aHUS is characterized by TMA and is distinct from other causes of TMA, such as Shiga toxin-induced HUS and thrombotic thrombocytopenic purpura, which is characterized by the reduction of ADAMTS13 activity. However, the renal histology and massive proteinuria did not fully support the initial diagnosis and indicated the coexistence of crescentic IgA nephropathy. Crescentic IgA nephropathy is an uncommon subtype accounting for ~ 5% of IgA nephropathy and has a poor prognosis [9]. Lv et al. [5] have reported that the initial serum creatinine level may predict kidney failure in patients with this disease and that patients with an initial serum creatinine level more than 6.8 mg/dL were less likely to recover from dialysis. An abnormal complement system may promote the development of IgA nephropathy [2]. In addition, the uncontrolled activation of the complement cascade is a significant etiological factor for the development of aHUS. Dysregulation of the complement system leads to endothelial cell, neutrophil, and platelet activation causing TMA which is associated with hemolytic anemia and thromobocytopenia, and may consequently cause severe damage to multiple vital organs including the kidneys [7]. Treatment options for aHUS include plasma exchange and the complement factor 5 inhibitor, eculizumab. The persistence of hemolysis or lack of improvement in renal function after 3 – 4 daily sessions of plasma exchange has been regarded as a criterion for uncontrolled aHUS and was suggested to be and is an indication for initiating eculizumab [1, 10]. Some investigators have reported that eculizumab is more effective in the early treatment of aHUS as it is able to protect the kidneys from ongoing complement-mediated damage and to allow the kidneys to recover from the reversible changes [11]. 

The Kidney Disease Improving Global Outcomes (KDIGO) guidelines recommend the use of steroids and cyclophosphamide to treat crescentic IgA nephropathy [12]. Recently, Ring et al. [13] have reported that eculizumab was effective in the treatment of a 16-year-old boy with the vasculitis form of IgA nephropathy who failed to respond to conventional aggressive therapy, including high-dose steroids, cyclophosphamide, and plasma exchange. This indicates that eculizumab may be effective in the treatment of crescentic IgA nephropathy as a last resort, because complement is possibly involved in the pathogenesis of IgA nephropathy. Rosenblad et al. [6] also reported that early initiation of eculizumab in patients with IgA nephropathy may have a beneficial effect by blocking complement-mediated renal inflammation. Wang et al. [2] have reported that the complement system abnormality might be associated with an increased risk for developing both aHUS and IgA nephropathy and could become a new target in the prevention of kidney injury. However, the use of eculizumab is still not included in any guidelines and there is not much data about its use in IgA nephropathy or C3 glomerulopathy. Furthermore, the high cost and the inconsistency about the duration of its use are still major challenges in the clinical settings. 

In the current case, eculizumab successfully ameliorated the hemolysis, but not the renal dysfunction, and the patient remained dialysis dependent. This might be in part due to the higher initial serum creatinine level (18.78 mg/dL) and the possible delay of treatment with eculizumab. This suggests that early diagnosis is important and that appropriate initiation of eculizumab may be needed in this type of AKI. 

## Acknowledgments 

The authors thank Professor Yoshihiko Ueda, Department of Pathology, Koshigaya Hospital, Dokkyo University School of Medicine, Saitama, Japan, for technical help with preparation and examination of renal histology. 

## Conflict of interest 

No support, financial or otherwise, was obtained for this work. 

**Table 1.** Serial changes of laboratory tests.

	Day 1	Day 3	Day 7	Day 14	Day 21	Day 28	Day 42	Day 56	Day 70
Hemoglobin (g/dL)	7.7	8.1	8.4	7.9	8.4	9.2	9.6	10.2	10.4
Platelet (104/μL)	5.4	7.4	9.8	10.3	11	11.8	12.6	15.3	17.7
CH50 (/mL)	22.2		23.7		25.7		32.2		43.1
Lactate dehydrogenase (IU/L)	1342	902	854	665	642	500	256	244	24
Total bilirubin (mg/dL)	4.5	3.2	2.4	1.6	0.8	0.5	0.4	0.4	0.4
D-dimer (μg/mL)	4.7		3.5		3.2		2.2		1.6
Creatinine (mg/dL)	18.78	12.35	11.93	10.54	9.52	8.88	7.72	7.65	7.22
Blood urea nitrogen (mg/dL)	121	67.3	36.9	49.2	33.3	32.8	33.8	44.2	54.4
Proteinuria (mg/dL)	570		363		220		210		166
Urinary liver type fatty acid binding protein (μg/g Cr)	273.2		156.8		98.8		71.2		28.2
Urinary N-acetyl-β-D-glucosaminidase (IU/L)	151				73				28
Urinary β2-microglobulin (μ/L)	5,008				1,420				584
